# Coronary microvascular dysfunction: a review of its association with extracardiac organ pathologies

**DOI:** 10.3389/fcvm.2025.1616332

**Published:** 2025-08-13

**Authors:** Yuting Duan, Jia Liu, Shan Wang, Tongxiao Luan, Jing Zhou, Ziwei Cui, Aohua Wang, Mengru Xu, Song Hu, Yongjun Mao

**Affiliations:** 1 Department of Geriatric Medicine, The Affiliated Hospital of Qingdao University, Qingdao, China; 2 Qingdao University, Laoshan District, Qingdao, Shandong, China

**Keywords:** coronary microvascular dysfunction, microvascular dysfunction, non-obstructive coronary artery disease, cardiovascular diseases, myocardial ischemia

## Abstract

Coronary microvascular dysfunction (CMD) is a high-risk factor for numerous cardiovascular events, and there is an increasing focus on the diagnosis and treatment of CMD itself or its association with cardiovascular diseases. However, some evidences suggest potential associations between CMD and multiple extracardiac pathologies, such as cerebrovascular, renal, pulmonary, retinal, hepatic, immune system diseases, and cancer. A shared pathological mechanism may underlie these connections, yet the relationship between CMD and other organs and systemic diseases remains unclear. Therefore, this review comprehensively assesses the current evidence base for the interplay between CMD and a range of systemic diseases, highlighting the need for a more integrated diagnostic and therapeutic approach.

## 1 Introduction

The coronary microcirculation is a blood circulation exchange system composed of anterior small arteries (500–100 μm), small arterioles (100–10 μm) and capillaries (<10 μm), is a critical regulator of myocardial perfusion, responsible for 80% of coronary vascular resistance (1). They work together to sustain the integrity of the coronary microcirculation and control the vascular tension and blood flow of the heart arteries (2). Coronary microvascular dysfunction (CMD), characterized by structural remodeling (e.g., capillary rarefaction, perivascular fibrosis) and functional impairment [e.g., reduced coronary flow reserve (CFR), endothelial dysfunction], has emerged as an independent predictor of adverse cardiovascular events, including myocardial ischemia, heart failure, and angina, even in the absence of obstructive coronary artery disease (3, 4). The detection method of CMD is shown in Supplementary Table S1.

Traditionally viewed as a cardiac-specific pathology, CMD is increasingly recognized as a systemic disorder. Recent evidence reveals its association with extracardiac organ pathologies, such as cerebrovascular disease, chronic kidney disease (CKD), retinal vascular dysfunction, liver disease, pulmonary arterial hypertension (PAH), autoimmune diseases, osteoporosis, sexual dysfunction, sarcopenia, obstructive sleep apnea (OSA), along with elevated morbidity and mortality among people living with human immunodeficiency virus (HIV), as well as increased incidence of cancer, which emphasize the prevalence of this important pathological mechanism. We analyzed and summarize the pathogenesis of CMD, review its association with other systemic diseases (Table 1). This review synthesizes current evidence on CMD systemic implications, emphasizing shared pathophysiological pathways and advocating for integrated diagnostic-therapeutic frameworks. We aim to redefine CMD as a multisystem disorder requiring collaborative clinical approaches.

**Table 1:** CMD associations with various organ systems and diseases.

Organ/system	Association with CMD	Specific mechanisms	Clinical research/findings
Brain	CMD is associated with CSVD, cognitive dysfunction, stroke and cerebral blood flow abnormalities.	– Common microvascular pathology (endothelial dysfunction, oxidative stress). – Abnormal coupling of brain-coronary hemodynamics	– 76% of CMD patients have cerebral hypoperfusion (5). – Patients with CMD have an increased risk of cognitive decline (C3 study, *n* = 67) (6).
Kidney	The prevalence of CMD is high among patients with CKD, which accelerates cardiovascular events	– Uremic toxin accumulation and oxidative nitrification stress – Bidirectional interaction of kidney-coronary microcirculation (hemodialysis and metabolic imbalance)	– A decrease in eGFR is associated with a decrease in CFR (*n* = 605) (7) – The CFR of dialysis patients was significantly decreased (hemodialysis vs control group) (8, 9)
Lung	The coexistence of PAH and CMD increases the risk of right heart failure.	– The reversal of the lung-coronary pressure gradient leads to insufficient coronary perfusion – Inflammation drives microvascular fibrosis	– The myocardial perfusion reserve index of patients with PAH was negatively correlated with pulmonary artery pressure (*r* = 0.79) (10). – 68% of patients with systemic sclerosis have CMD (*n* = 120) (11).
Retina	Retinal microvascular abnormalities can predict CMD, with significant gender specificity.	– Endothelial dysfunction (NO↓, ET-1↑). – Inflammatory factors (IL-6, TNF-α) induce the shedding of glycocalyx	– Retinal arteriolar stenosis is associated with reduced myocardial perfusion (*β* = 0.0088, *P* = 0.04) (12). – The reduction in arteriolar calibre of females is a better predictor of CHD mortality than that in males (13).
Liver	Non-alcoholic fatty liver disease (NAFLD) is closely related to CMD and independent of traditional risk factors.	– Liver fibrosis impairs coronary reactivity through the inflammation-metabolic axis – Insulin resistance inhibits myocardial glucose uptake	– The coronary flow velocity reserve of patients with NAFLD was significantly decreased (14). – The liver fibrosis score is negatively correlated with myocardial perfusion reserve (15).
Immune System Diseases	Autoimmune diseases (such as SLE, RA, and psoriasis) significantly increase the risk of CMD.	– Autoantibodies (such as anti-β 2GPI) activate the TLR4 pathway – The deposition of extracellular traps (NETs) of neutrophils reduces microvessel density	– The CFR of rheumatoid arthritis patients decreased (Meta-analysis, *n* = 709) (16). – 31.5% of patients with psoriasis have CMD (*n* = 448) (17).
Cancer	The bidirectional association between CMD and cancer: CMD increases the risk of cancer, and anti-cancer treatment aggravates microvascular damage.	– Chronic inflammation is jointly driven by oxidative stress – Anti-cancer drugs (such as sunitinib) directly damage microvessels	– Cancer risk of CMD patients ↑ (HR = 4.91, Breast cancer cohort) (18). – The CFR of patients treated with sunitinib was significantly reduced (*n* = 18) (19).
Others	Metabolic syndrome, osteoporosis, sexual dysfunction interact with CMD.	– High PTH levels impair endothelial function – Inflammation and vascular remodeling related to obstructive sleep apnea (OSA)	– CMD reversal after parathyroidectomy (*n* = 100) (20). – Severe OSA is associated with CMVO after STEMI (*n* = 249) (21).

CMD, coronary microvascular dysfunction; CSVD, cerebral small vessel disease; CKD, chronic kidney disease; CFR, coronary flow reserve; PAH, pulmonary arterial hypertension; CHD, coronary heart disease; NAFLD, non-alcoholic fatty liver disease; SLE, systemic lupus erythematosus; RA, rheumatoid arthritis; CHD, coronary heart disease; PTH, Parathyroid hormone; OSA, obstructive sleep apnea; CMVO coronary microvascular dysfunction and obstruction; STEMI, ST-segment elevated myocardial infarction.

## 2 Key pathophysiological mechanisms

**Figure 1:**
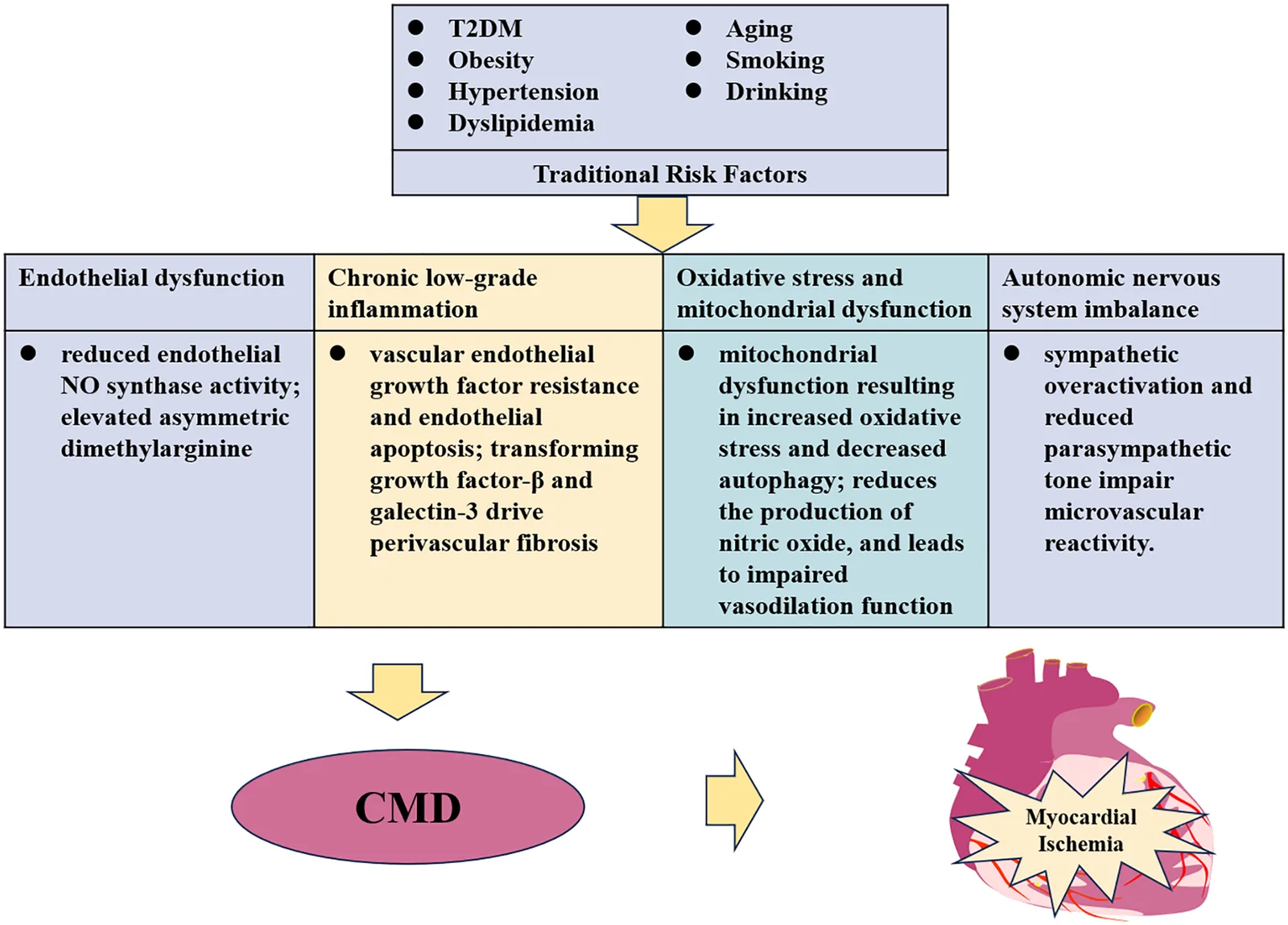
Pathophysiological mechanisms linking traditional risk factors to coronary microvascular dysfunction (CMD) and myocardial ischemia. This schematic illustrates the multifactorial pathogenesis of CMD. Traditional cardiovascular risk factors [e.g., type 2 diabetes mellitus (T2DM), obesity, hypertension, dyslipidemia, aging, smoking, and alcohol consumption] converge to drive four core pathological mechanisms: Endothelial dysfunction: Characterized by reduced nitric oxide (NO) bioavailability due to impaired endothelial NO synthase (eNOS) activity and elevated asymmetric dimethylarginine (ADMA). Chronic low-grade inflammation: Mediated by vascular endothelial growth factor (VEGF) resistance, endothelial apoptosis, and perivascular fibrosis driven by transforming growth factor-β (TGF-β) and galectin-3. Oxidative stress and mitochondrial dysfunction: Results in excessive reactive oxygen species (ROS) production, impaired autophagy, and diminished vasodilatory capacity. Autonomic nervous system imbalance: Sympathetic overactivation and reduced parasympathetic tone impair microvascular reactivity. These interconnected mechanisms collectively contribute to CMD, ultimately leading to myocardial ischemia. Arrows indicate the progression from risk factors to end-organ damage.

The development of CMD is a complex process (Figure 1), and conventional risk factors for cardiovascular disease, such as type 2 diabetes mellitus, obesity, hypertension, and dyslipidemia, may contribute to its pathogenesis (22). Additionally, the mechanisms behind CMD include (a) Endothelial dysfunction: Endothelial dysfunction is a hallmark of CMD, driven by impaired nitric oxide (NO) bioavailability and oxidative stress. Reduced endothelial NO synthase (eNOS) activity, coupled with increased reactive oxygen species (ROS) production (e.g., via NADPH oxidase), disrupts the balance between vasodilators (NO, prostacyclin) and vasoconstrictors (endothelin-1, angiotensin II) (23, 24). Recent studies implicate asymmetric dimethylarginine (ADMA), an endogenous eNOS inhibitor, as a key mediator in conditions like diabetes and CKD, where elevated ADMA levels correlate with impaired CFR (25). (b) Chronic low-grade inflammation: Chronic hypoxia and inflammation trigger capillary dropout through vascular endothelial growth factor resistance and endothelial apoptosis (via caspase-3 activation) (26, 27). Concurrently, transforming growth factor-β and galectin-3 drive perivascular fibrosis, increasing extracellular matrix stiffness and impairing vasodilatory capacity (28). (c) Oxidative stress and mitochondrial dysfunction: Mitochondria cause changes in cell metabolism and respiration, and produce excessive ROS, resulting in increased oxidative stress and decreased autophagy, damages endothelial cells, makes them release inflammatory factors and adhesion molecules, promotes white blood cell adhesion and migration, and leads to vascular inflammation. At the same time, oxidative stress destroys the antioxidant system of endothelial cells, reduces the production of NO, and leads to impaired vasodilation function (29, 30). (d) Autonomic nervous system imbalance: Sympathetic overactivation (e.g., via α₁-adrenergic receptors) and reduced parasympathetic tone impair microvascular reactivity. Elevated norepinephrine levels in heart failure patients correlate with reduced CFR, while β-blockers partially restore microvascular function by attenuating adrenergic-driven vasoconstriction (31).

## 3 Coronary microvascular dysfunction in relation to other systems and organs

### 3.1 Brain-heart vascular interconnections

The brain and heart exhibit striking similarities in vascular architecture, characterized by intricate networks of conduit arteries traversing the organ surface to deliver blood through dense capillary beds. Both organs require precise regulation of local perfusion to meet high metabolic demands. Importantly, they share overlapping risk factors for microvascular dysfunction, including aging, hypertension, diabetes mellitus, smoking, systemic inflammation, vasospasm, and microemboli, which collectively contribute to the pathogenesis of CMD and cerebral small vessel disease (CSVD) (32, 33). Structural and functional abnormalities in the microcirculation serve as the common pathological substrate for both conditions (34).

#### 3.1.1 Emerging evidence from clinical studies

Clinically, CMD manifests not only as angina but also with extracardiac features such as pathological pain perception, migraine-like symptoms, and autonomic dysregulation (35–37). Conversely, CSVD accounts for 25% of ischemic strokes and 45% of dementia cases, presenting with diverse neurological deficits including facial nerve palsy, gait disturbances, aphasia, and subcortical white matter lesions evident on neuroimaging (38, 39). Despite these clinical parallels, the mechanistic links between CMD and CSVD remain incompletely elucidated. Targeted research is urgently needed to unravel their interplay and mitigate the combined disease burden. The prospective Cerebral Coronary Connection (C3) study provided critical insights by examining 67 patients with blinded assessments. Mejia et al. demonstrated that CMD correlates with CSVD severity, cerebral hemodynamic impairment, and accelerated cognitive decline, underscoring its systemic implications beyond the coronary circulation (6). Supporting this, a clinicopathological analysis of 175 dementia patients by Andin et al. revealed that small-vessel dementia subtypes exhibit a higher prevalence of cardiovascular lesions compared to other dementia etiologies (40). Neuroimaging studies further strengthen this association: 76% of 95 CMD patients exhibited cerebral hypoperfusion on perfusion scans (5), while Pai et al. identified a strong concordance between cardiac perfusion defects and cerebral hypoperfusion lesions in CMD cohorts (41). Similar findings were replicated by Sun and colleagues, suggesting a shared hemodynamic vulnerability (42).

#### 3.1.2 CADASIL: a paradigm of systemic microangiopathy

Cerebral autosomal dominant arteriopathy with subcortical infarcts and leukoencephalopathy (CADASIL), caused by NOTCH3 mutations, exemplifies the systemic nature of microvascular dysfunction. Although classically defined by early-onset stroke and leukoencephalopathy, CADASIL is increasingly recognized as a multisystem disorder. Argiro et al. conducted a case-control study comparing 17 CADASIL patients with 15 healthy controls, revealing significantly reduced CFR indicative of CMD in the CADASIL group (43). Coronary angiography in a 45-year-old CADASIL patient by Rubin et al. demonstrated diffuse left anterior descending artery stenosis and histopathological features resembling CMD, despite the absence of traditional atherosclerosis (44). Mechanistically, CADASIL-related CMD arises from vascular fibrosis, impaired endothelial autoregulation, and heightened myogenic tone—pathways that may overlap with sporadic microvascular disease (45).

### 3.2 Kidney-heart microvascular crosstalk

Emerging evidence positions CMD as a pivotal mediator linking CKD to cardiovascular morbidity. Notably, cardiovascular complications frequently manifest in CKD patients even in the absence of obstructive coronary artery disease, implicating microcirculatory pathology as a key determinant (46).

#### 3.2.1 Clinical evidence of renal-coronary interaction

A seminal study by Bajaj et al. (*n* = 352) integrating renal functional assessment, cardiac positron emission tomography (PET), and echocardiography demonstrated that CMD accounts for 58% of the association between renal impairment, myocardial dysfunction, and cardiovascular events. This finding highlight CMD-targeted therapies as potential strategies for mitigating cardiovascular risk in uremic cardiomyopathy (47). In 605 patients stratified by renal function, reduced estimated glomerular filtration rate (eGFR) correlated with diminished CFR, which may suggest parallel alterations in renal and coronary microcirculation early in the course of the disease (7). A prospective cohort of 175 CKD patients revealed stage-dependent CFR deterioration, with proteinuria serving as an independent predictor of microvascular impairment (48).

#### 3.2.2 Dialysis modality and transplantation considerations

While retrospective PET analysis of 435 stage 1–3 CKD patients showed comparable CFR to controls after adjusting for cardiovascular risks (49), end-stage renal disease exhibits distinct patterns: a study of dialysis patients and controls revealed that patients in the hemodialysis (HD) group had a lower CFR compared to controls (8, 9). There was no difference in CFR between HD and peritoneal dialysis patients, but it was significantly lower in diabetic patients and those with more severe diastolic dysfunction (50). Studies on the impact of renal transplantation on CFR are limited. Although renal function is partially restored after renal transplantation, the incidence of CMD after renal transplantation remains high, with 8%–65% of patients still having a CFR < 2 (51, 52).

#### 3.2.3 Pathophysiological mechanisms

The CKD-induced microvascular insult arises through synergistic pathways: (a) Hemodynamic Stressors: Pressure overload from hypertension and arterial stiffness elevates pulse wave velocity. Volume overload secondary to anemia and hyperthyroidism augments preload. These forces culminate in capillary bed shear stress and rarefaction, establishing a bidirectional link between microvascular dysfunction and eGFR decline (53). (b) Metabolic and Inflammatory Drivers: Uremic toxin accumulation, aldosterone system activation, and hyperuricemia promote myocardial fibrosis. Oxidative-nitrosative stress and chronic inflammation accelerate microvascular endothelial apoptosis. These processes collectively drive coronary microcirculatory remodeling and elevate cardiovascular mortality (54).

### 3.3 Lung-heart microvascular interdependence

PAH characterized by elevated pulmonary vascular resistance and right ventricular afterload, represents a critical intersection of pulmonary and coronary microcirculatory dysfunction. Chronic pulmonary hypoperfusion induces right coronary artery compression and microvascular ischemia, driving progressive ventricular remodeling that culminates in right heart failure—the leading cause of PAH-related mortality (55).

#### 3.3.1 Clinical evidence of cardiopulmonary microangiopathy

PAH is a common complication of systemic sclerosis (SSc), and patients with SSc have systemic endothelial dysfunction associated with disease severity (56). Emerging data position PAH as a systemic microvascular disorder: (a) Connective Tissue Disease Context: In SSc, endothelial dysfunction propagates across vascular beds. A study of 120 SSc patients by Komócsi et al. revealed 42% concurrent PAH, 31% coronary artery disease, and 68% reduced CFR, demonstrating tri-vascular bed involvement, the findings revealed considerable overlap between PAH, CAD and reduced CFR in patients with SSc (11). Pressure-Perfusion Coupling: In 25 PAH patients (Jens et al.) showed myocardial perfusion reserve index inversely correlating with pulmonary artery pressure (*r* = −0.79, *P* < 0.002), suggesting pressure-mediated microvascular rarefaction (10). Harris et al.'s population study (*n* = 3,397) established lung-kidney-retina-heart microvascular synergy: retinal venular diameter was negatively correlated with forced expiratory volume in the first second (FEV₁) and FEV₁/ forced vital capacity (FVC) (*P* < 0.001 and *P* = 0.04). The albumin-to-creatinine ratio was negatively correlated with FEV₁ (*P* = 0.002) but not with FEV₁/FVC. Myocardial blood flow (*n* = 126) was associated with lower FEV₁, lower FEV₁/FVC, and higher LAA percentage (*P* = 0.02, *P* = 0.001, and *P* = 0.04) (57). These cross-sectional data raise the possibility that lung dysfunction may be a component of systemic microvascular disease, that lung damage may be related to end-organ failure in all circulations, or that there may be a shared susceptibility.

#### 3.3.2 Pathophysiological pathways in PAH-induced ischemia

The ischemic cascade in PAH arises through multifactorial mechanisms:(a) Biomechanical Stressors: Right ventricular dilation increases intramural tension, elevating myocardial oxygen demand by 35%–40% while simultaneously impairing subendocardial perfusion (58). Pulmonary-to-systemic pressure gradient reversal reduces coronary driving pressure during diastole, the critical perfusion phase. (b) Neurohormonal Activation: Compensatory sympathetic overdrive and renin-angiotensin-aldosterone system activation promote eNOS uncoupling. Catecholamine excess induces microvascular *α*1-adrenergic hyperreactivity and vasospasm. (c) Microvascular Remodeling: SSc-associated pericyte apoptosis and capillary dropout reduce vascular compliance. Uremic toxin accumulation (in PAH-renal syndrome) accelerates glycocalyx shedding, increasing leukocyte adhesion by 3-fold (58).

### 3.4 Retinal microvasculature as a coronary microvascular surrogate

Emerging evidence reveals structural and functional parallels between retinal and coronary microcirculation, suggesting pan-vascular endothelial dysfunction as their common substrate. Retinal vascular metrics may serve as non-invasive biomarkers for coronary microvascular disease.

#### 3.4.1 Clinical evidence of retinal-coronary coupling

(a) Hemodynamic Correlates: in CMD patients, impaired coronary slow flow phenomenon correlates higher retinal arteriolar flow velocity (*r* = −0.405, *P* = 0.03), reflecting compensatory hyperemia from defective vasodilation (59). An analysis of 212 non- coronary artery disease (CAD) subjects demonstrated that retinal arteriolar stenosis predicts reduction in myocardial perfusion (*β* = 0.0088, *P* = 0.04), though attenuated after adjusting for traditional risk factors (*β* = 0.0037, *P* = 0.33) (12). (b) Sex-Specific Vulnerability: It is acknowledged that CMD has a significant role in coronary heart disease (CHD). Women may be more susceptible to the development of CHD than males are because they frequently exhibit the symptoms of the condition without having obstructive CAD (60). Meta-analysis of 22,159 individuals revealed gender-dimorphic associations: female: Narrow retinal arterioles (HR = 1.17, *P* = 0.02) and venular caliber (HR = 1.16, *P* = 0.03) predict CHD; male: No significant association (HR = 1.02, *P* = 0.17). This provides additional evidence supporting the use of retinal vessel diameter changes as a predictor of CHD (61). Population study (*n* = 3,654) confirmed each SD decrease in arteriolar calibre predicted a 1.3–2-fold higher risk of CHD death in women, but it is lower in men, supporting the hypothesis that microvascular disease may be more prevalent in women with CHD (13). (c) Retinopathy as Systemic Marker: Diabetic retinopathy associated with a twofold higher risk of incident CHD events (HR = 2.07, 95% CI: 1.38–3.11) and a threefold higher risk of fatal CHD (HR = 3.35, 95% CI: 1.40–8.01), Non-diabetic retinopathy carries comparable risk (HR = 2.16, 95% CI: 1.16–4.02), independent of glucose metabolism (62, 63).

#### 3.4.2 Pathophysiological links

The retinal-coronary microvascular axis shares key pathological pathways: (a) Endothelial dysregulation: Reduced nitric oxide bioavailability and increased endothelin-1 impair flow-mediated dilation in both beds. (b) Inflammatory Crosstalk: IL-6 and TNF-α induce glycocalyx shedding, elevating leukocyte adhesion by 3.8-fold in retinal and coronary microvessels. (c) Structural Remodeling: Media-to-lumen ratio increasedriven by chronic shear stress (64, 65).

### 3.5 Hepato-cardiac metabolic-vascular axis

The relationship between liver and heart pathophysiology is becoming more and more significant. A growing focus has been placed on less severe but more prevalent liver illnesses as a result of changing demographics. One such condition is metabolic fatty liver disease, which has an estimated global prevalence of 25% (66). Although severe liver diseases like cirrhosis or hepatocellular carcinoma can result from the fatty liver phenotype, cardiovascular disease is the main cause of death for individuals (67).

#### 3.5.1 Clinical evidence of liver-coronary crosstalk

(a) Fibrosis-Microvascular Coupling: A study (*n* = 66) demonstrated liver fibrosis (fibrosis-4 risk score) specifically associates with lower myocardial perfusion reserve (*β* = −1.12, *P* = 0.02), independent of traditional cardiovascular disease (CVD) risk factors (15). Doppler analysis of 24 non-alcoholic fatty liver disease (NAFLD) vs. 28 controls revealed severely impaired coronary flow velocity reserve (CFVR: 1.65 ± 0.36 vs. 2.67 ± 0.81, *P* < 0.001), indicating pressure-independent microvascular compromise (14). Targher et al. pointed out in their authoritative review that MASLD (formerly NAFLD) is a systemic metabolic disorder centered on insulin resistance and is significantly associated with CMD and CVD risk. Epidemiological studies show that: The cardiovascular mortality rate in MASLD patients is as high as 4.2 per 1,000 person-years, far exceeding liver disease-related mortality (0.92 per 1,000 person-years), and advanced liver fibrosis further amplifies CVD risk (HR = 2.50, 95% CI: 1.68–3.72) (68); (b) Metabolic Dysregulation: Lee et al, in their study of 131 patients with type 2 diabetes, found that subjects with NAFLD demonstrated significantly decreased myocardial glucose uptake (*P* = 0.018) (69). Meanwhile, abdominal wall fat index was significantly related to CFVR (*r* = −0.46, *P* = 0.011) and insulin resistance (*r* = −0.71, *P* < 0.0001). CFVR could be noninvasively evaluated using transthoracic doppler echocardiography (TTDE). Coronary endothelial dysfunction indicated as CFVR, body fat distribution and insulin resistance was quantitatively correlated in obesity (70). A retrospective cohort study involving a total of 886 patients, with data spanning from 2006 to 2014, found that compared to non-NAFLD patients, NAFLD patients had a higher prevalence of coronary microvascular dysfunction (64.8% vs. 43.4%; *P* < 0.001) and lower CFR (1.9 ± 1.1 vs. 2.2 ± 0.7; *P* < 0.001). Additionally, NAFLD independently predicted coronary microvascular dysfunction (*P* = 0.01) (71). Lautamäki et al. studied 55 patients with type 2 diabetes and coronary artery disease using PET and proton magnetic resonance spectroscopy (¹H-MRS). They found that patients with high liver fat content (>8%) had more severe myocardial insulin resistance and coronary artery dysfunction. Specifically, the high liver fat group had significantly lower myocardial glucose uptake (*P* = 0.040), and their CFR was 28% lower than the low liver fat group (*P* = 0.02), with a negative correlation between liver fat content and CFR (*r* = −0.38, *P* = 0.020). Additionally, high liver fat patients had elevated high-sensitivity C-reactive protein (hsCRP) and soluble adhesion molecules (e.g., E-selectin, VAP-1), suggesting low-grade inflammation may exacerbate CMD via endothelial dysfunction. This study first confirmed that liver fat content is an independent predictor of myocardial insulin resistance and coronary microvascular function, highlighting NAFLD's key role in cardiovascular metabolic disorders (72). Hepatic small extracellular vesicles contribute to endothelial hyperpermeability in coronary microvessels by delivering novel-miR-7 and targeting the LAMP1/Cathepsin B/NLRP3 inflammasome axis during NAFLD (73).

#### 3.5.2 Pathophysiological mechanisms

Low-grade inflammation, lipotoxicity, oxidative stress and severe impairments to insulin sensitivity, coronary artery function, and myocardial glucose uptake can result from asymptomatic excessive liver fat accumulation. Reduced coronary vascular reactivity, thrombosis, and fibrosis are additional consequences of inflammation that are linked to the onset of endothelial dysfunction and unfavorable cardiac remodeling mechanisms. During ischemia, impaired myocardial glucose uptake is harmful to the myocardium. Atherosclerotic lesion growth at an accelerated pace, plaque vulnerability, and insulin resistance are all impacted by poor insulin signaling and insulin resistance. Our group developed propylene glycol alginate sodium sulfate nanoparticles (PSS-NP) to target diabetic microangiopathy. In a rodent model of diabetic cardiomyopathy, PSS-NP effectively restored coronary microvascular function by simultaneously improving endothelial health, suppressing pro-coagulant PAI-1, and mitigating oxidative stress via the AGEs/RAGE/NF-κB axis—highlighting a promising multi-target approach for metabolic CMD (74).

### 3.6 Immune-microvascular crosstalk in coronary dysregulation

Various immune system diseases can cause microvascular dysfunction in the coronary microcirculation. Studies have demonstrated that autoimmune rheumatic diseases (ARDs), such as systemic lupus erythematosus (SLE), rheumatoid arthritis (RA), systemic vasculitis, spondyloarthropathies (e.g., psoriatic arthritis), and SSc, are associated with an increased risk of cardiovascular events (17, 75, 76).

#### 3.6.1 Clinical spectrum of immune-mediated CMD

(a) Autoimmune rheumatic diseases: In a study of 207 CMD patients, women with a history of ARDs had a worsened myocardial perfusion reserve and a lower functional cardiac condition and state (77). Patients with ARDs may be more likely to experience significant adverse cardiac events linked to CMD even in the absence of obstructive CAD (78). Similarly, a meta-analysis that included 709 patients with rheumatic diseases and 650 controls found that patients with rheumatic diseases had significantly lower CFR (16). (b) Rheumatoid Arthritis: A 5-year follow-up study of SLE patients showed that nearly half had myocardial perfusion similar to or worse than CMD without obstructive CAD, this also supports CMD as a cause of cardiac morbidity and mortality in SLE (79). (c) Rheumatoid Arthritis: Recio-Mayoral et al. found that PET showed CMD in RA patients without common cardiovascular risk factors or epicardial CAD (80). A meta-analysis of 41,490 cases showed a 48 percent increase in cardiovascular risk in people with RA compared with people without RA (81). Notably, coronary and peripheral microvascular dysfunction has been observed in early RA, even 6 months after initial diagnosis (82). Weber et al, in their study of patients with systemic vasculitis, found that patients with vasculitis also had more frequent and severe CMD, supporting the potential role of inflammation in driving coronary vasodilatory abnormalities (83).(d) Systemic Sclerosis: similarly, patients with SSc or psoriasis had worse microvascular function compared with healthy controls (84–86). Nitenberg et al. studied patients with primary scleroderma cardiomyopathy and healthy controls and found that the scleroderma group had significantly reduced coronary flow reserve in the absence of significant coronary artery stenosis (87). Maurizio et al. included 20 patients with diffuse SSc without signs or symptoms of CVD and 20 age- and sex-matched controls in their study and found that CFR was also significantly lower in patients with SSc (*P* = 0.0033) (88). In another study, 31.5% of 448 patients with psoriasis developed CMD, which was associated with psoriasis severity and duration, suggesting that systemic inflammation may play a role in CMD and that the coronary microcirculation may be an extracortical site involved in immune-mediated damage in psoriasis, and that patients with severe psoriasis should be promptly diagnosed and actively screened for CMD (17). (e) Others: A study of 37 patients with inflammatory bowel disease and 30 controls found that 40% of inflammatory bowel disease patients had CMD (89). Furthermore, it is well established that endothelial dysfunction and inflammation are risk factors associated with HIV that may facilitate the pathogenesis of CMD. In HIV-positive individuals without a history of cardiovascular illness, coronary microvascular changes should receive more attention since they may be significant mediators of subclinical myocardial dysfunction (90).

#### 3.6.2 Pathogenic triad in immune-mediated CMD

(a) Cytokine Storm Effects: Therapies for immune system disorders, such as biologic therapies, may affect the coronary microcirculation. Prospective cohort studies have shown significant reductions in coronary inflammation and total plaque burden in patients receiving biologic therapies, which may affect coronary vasodilatory function (91, 92). In general, the pro-inflammatory nature of ARDs may impede normal cardiac blood flow regulatory mechanisms and coronary microvascular function, increasing the risk of myocardial ischemia and long-term cardiovascular events (93). (b) Autoantibody Arsenal: Patients with rheumatoid arthritis have higher titers of autoantibodies against the human leukocyte antigen/beta-2-glycoprotein I complex (94, 95). Anti-beta-2-glycoprotein I complex activate TLR4/MyD88 pathway, increasing coronary thrombogenicity (96, 97). (c) Vasculitic Remodeling: Neutrophil extracellular traps (NETs) deposit in vasa vasorum, reducing microvascular density (98, 99).

### 3.7 Onco-cardiac microvascular nexus

Emerging paradigms position CMD as both a precursor and consequence of malignancy, forming a self-perpetuating cycle through shared inflammatory-metabolic pathways (18, 100, 101).

#### 3.7.1 Clinical evidence of bidirectional coupling

(a) Cancer—CMD axis: Divakaran et al. investigated the association between CMD, as measured by PET, and adverse cardiovascular events in a cohort of 87 breast cancer patients without clinically significant CAD. The study found that coronary artery diastolic dysfunction was linked to cardiovascular events in patients with breast cancer (HR: 4.91; 95% CI: 1.68–14.38; *P* = 0.004) (18). Previous studies have shown a bidirectional association between cancer and CAD (102). In a cross-sectional study, 18 patients with metastatic cancer who were treated with sunitinib and 27 healthy subjects were included. The study found that cancer patients treated with sunitinib had significantly impaired CFR. The duration of sunitinib treatment and inflammatory markers were negatively correlated with CFR (19). (b) CMD-Oncogenesis axis: Rajai et al. investigated the relationship between CMD and cancer in a cohort of patients with angina and non-obstructive CAD using invasive measurements of CFR. They discovered that the presence of CMD was linked to an elevated risk of cancer (103). The left anterior descending artery's CFVR, which is measured during high-dose vasodilator stress echocardiography, examines both the microcirculatory and epicardial functions of the heart. It has been found to be negatively correlated with microvascular dysfunction, chronic inflammation, and obstructive coronary artery disease. In their study of 1,002 patients undergoing Transthoracic Doppler echocardiography, Gaibazzi et al. found that a decrease in CFVR was independently associated with cancer deaths (104).

#### 3.7.2 Pathobiological interplay

Additionally, regardless of whether the cancer treatment is physical, chemical, hormonal, or biological, it may have adverse effects on the cardiovascular system. The treatment of cancer causes myocardial ischemia through various mechanisms, including accelerated atherosclerosis, thrombosis, vasospasm, and damage to the coronary microvasculature (105). (a) Impaired microcirculation: Impaired microcirculation may contribute to a pro-cancer environment by activating angiogenic pathways that promote tumor growth. Recent studies have shown that patients with a history of cardiovascular disease are at an increased risk of developing cancer (104). (b) Inflammatory feedforward Loop: Chronic inflammation and oxidative stress may serve as shared pathways linking CMD to cancer progression (106, 107). In the treatment of testicular cancer, cisplatin is activated through the renin-angiotensin-aldosterone system, intensifying the promotion of significant inflammation by activating the transcription factor NF-κB, stimulating the expression of cell adhesion molecules and releasing pro-inflammatory cytokines (such as IL-1, IL-6 and TNF-α) (108, 109). (c) Oxidative stress: The anti-cancer drug doxorubicin (DOX) is toxic to target cells, but it can also cause endothelial dysfunction and edema secondary to oxidative stress in the vascular wall. After being exposed to the clinically relevant concentration of DOX (up to 1 micron) for 24 h, the permeability of bovine pulmonary artery endothelial cells monolayer to albumin increased by approximately 10 times compared with the control group (110).

### 3.8 Other

Prasad et al. conducted a study which found that postmenopausal women with CMD had twice the likelihood of developing osteoporosis after a 7-year follow-up period (111). In a study conducted by Reriani et al, the development of erectile dysfunction (ED) was assessed in 130 patients with coronary atherosclerosis but without severe stenosis. This was done through a questionnaire after a mean follow-up of 8.4 years. The study found that CMD was a predictor of ED development in men with coronary atherosclerosis but without severe stenosis (112). In their follow-up study of 400 patients (median 6.0 years), Souza et al. found that muscle deficiency, rather than excessive obesity, was independently associated with CMD and poor future outcomes, particularly heart failure (113). Elena et al. analyzed 100 patients with primary hyperparathyroidism (PHPT) and 50 control patients. According to the study, CMD is fully restored in PHPT patients following parathyroidectomy. Parathyroid hormone (PTH) has an independent correlation with CMD, indicating that the hormone plays a critical role in elucidating the elevated cardiovascular risk observed in PHPT (20). OSA is a risk factor for cardiovascular disease. Patients with OSA may develop CMD due to increased levels of inflammatory factors, vascular remodeling caused by changes in transmural pressure, smooth muscle cell hypertrophy, and endothelial dysfunction (114). In a study conducted by Yoshitaka et al. on 249 patients with ST-segment elevated myocardial infarction (STEMI) who underwent their first percutaneous coronary intervention, it was found that coronary microvascular dysfunction and obstruction (CMVO) was a significant factor in predicting poor outcomes, and severe OSA is associated with CMVO in patients with STEMI (21).

## 4 Conclusion

**Figure 2:**
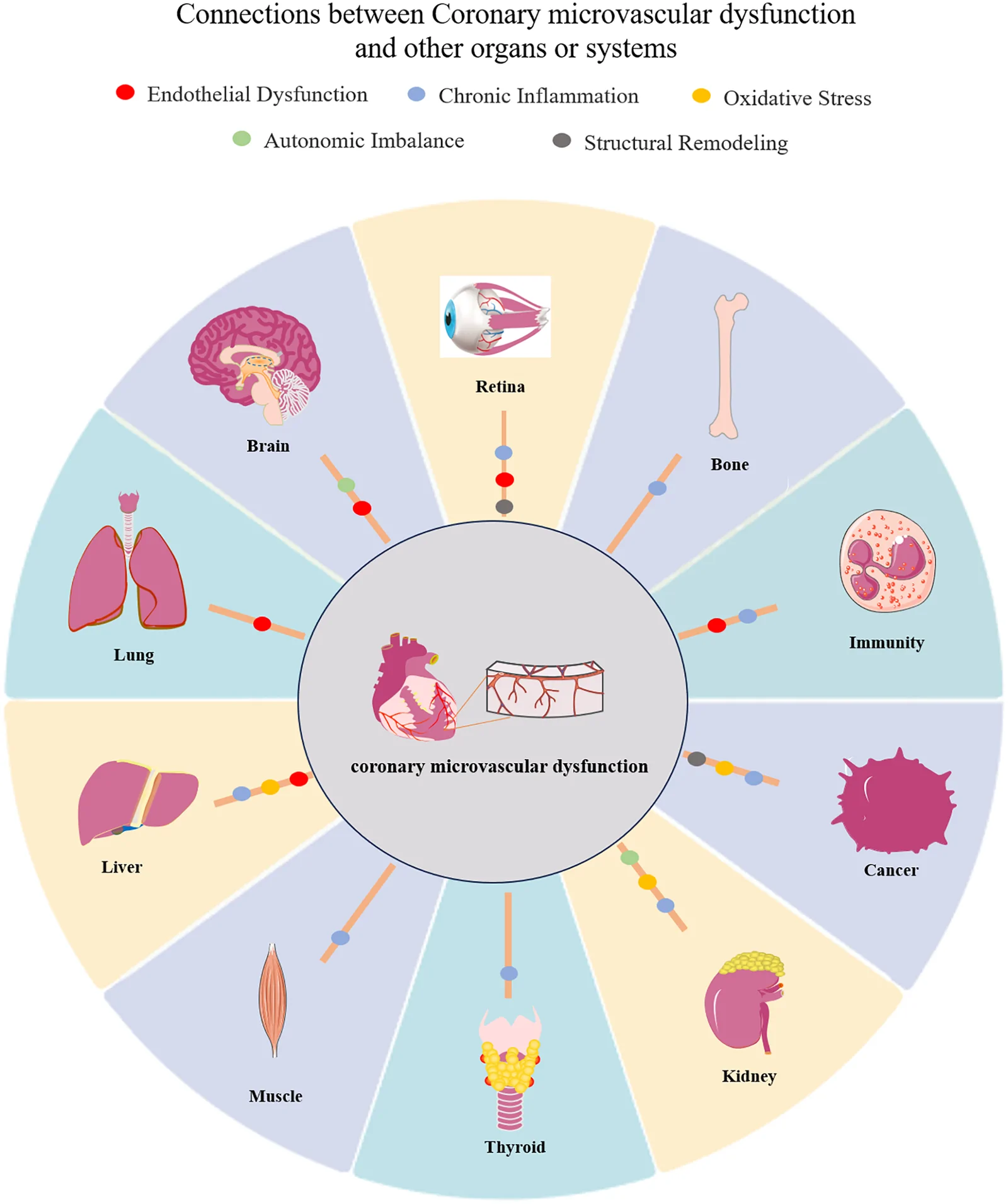
Systemic interconnections between coronary microvascular dysfunction (CMD) and extracardiac organ pathologies. CMD (central node) propagates multiorgan damage through five shared pathological pathways: endothelial dysfunction, chronic inflammation, oxidative stress, autonomic imbalance, structural remodeling. These mechanisms bidirectionally link CMD to dysfunction in 11 major organ systems: brain, retina, kidney, lung, liver, immune system, cancer, muscle, bone, thyroid, vascular network. Arrows denote bidirectional pathological crosstalk. See Table 1 for detailed clinical evidence.

Coronary microvascular dysfunction (CMD) transcends its traditional cardiac confinement, emerging as a systemic disorder intricately linked to multiorgan pathologies (Figure 2). The microvascular system is crucial for maintaining the homeostasis of the heart and other organs. In certain diseases, it is important to consider not only the disease itself but also other microvascular lesions to prevent subsequent complications. Although the clinical relevance provides a framework for understanding the systemic impact of CMD, the causal mechanisms of CMD in relation to other systems have not yet been fully verified. There are significant knowledge gaps regarding many disease-combined CMD, these gaps include mechanisms by which other diseases affect CMD pathophysiology, the role of disease therapeutic agents on CMD and cardiovascular outcomes, upgrades to both invasive and non-invasive approach methods, as well as standardization of protocols, and optimal treatment of microvascular and vasospastic angina. Future prospective studies need to address these issues in risk assessment to improve the quality of life of patients with comorbidities and reduce disease complications.
