# Supplementary material for: Coronary microvascular dysfunction: a review of its association with extracardiac organ pathologies
Source: Front Cardiovasc Med. 2025 Aug 13;12:1616332. doi: 10.3389/fcvm.2025.1616332 (PMC12380738; doi:10.3389/fcvm.2025.1616332)
Supplement: Supplementary file 1 [file Table1.docx]

Supplementary Table 1: Coronary microvascular dysfunction main tests.

| Imaging modality | Imaging technique | Substance | Diagnostic threshold |
| --- | --- | --- | --- |
| **Noninvasive** |  |  |  |
| TTDE | Pulsed-wave Doppler on the proximal LAD artery | Adenosine Dipyridamole | CFRV < 2 |
| BTD | angiography images and blood flow velocity | _ | caIMR>25 |
| MCE | Perfusion score and myocardial blood volume | contrast agents | MBF < 2 |
| PET | Dynamic first-pass vasodilator stress and then  rest perfusion imaging | Radioisotope tracers–^13^N-ammonia, ^82^Rb, Adenosine | MPR < 2 |
| CMR | Dynamic first-pass vasodilator stress and then rest perfusion imaging | Gadolinium-based contrast agents Vasodilators | MPRI < 2 |
| CT scan | Resting and stress perfusion imaging | Adenosine, iodine-based contrast agents | MPR < 2 |
| **Invasive** |  |  |  |
| Coronary angiography | Dynamic passage of angiographic contrast | Iodine-contrast agents | TIMI–2 TFC > 25frames |
| Provocation spasm test (ACH/Erg) | Coronary vascular channels | Acetylcholine, ergonovine | ≤1.5 |
| Intracoronary thermodilution | Estimation of coronary blood flow using bolus (mean transit time) or continuous thermodilution techniques | Adenosine, Papaverine Saline solutions | CFR < 2–2.5  IMR > 25 |
| Intracoronary Doppler flow-pressure wire | Direct measure of the coronary peak flow velocity | Adenosine | CFR < 2.5  HMR > 1.7 |
| Intracoronary vasoreactivity testing | Intracoronary infusion of vasoactive agents | Acetylcholine Ergonovine | ECG asocontraction on angiography Symptoms |

Continued table 1

| Bolus thermodilution | Pressure-Temperature Sensor Wire | Adenosine | RRR < 3.5 |
| --- | --- | --- | --- |
| Continuous thermodilution Bolus thermodilution Doppler | Pressure-Temperature Sensor Wire | Adenosine | MRR-No formal cut-off points  established |

TTDE, transthoracic doppler echocardiography; CFRV, coronary ﬂow reserve velocity; BTD, bolus thermodilution; IMR, index of microcirculatory resistance; MCE, myocardial contrast echocardiography; MBF, myocardial blood ﬂow; PET, positron emission tomography; MPR, myocardial perfusion reserve; CMR, cardiac magnetic resonance; MPRI, myocardial perfusion reserve index; LAD, left anterior descending; TIMI, thrombolysis in myocardial infarction; TFC, TIMI frame count; ACH, Acetylcholine; Erg, ergonovine; CFR, coronary flow reserve; HMR, hyperemic microvascular resistance; ECG, electrocardiography; RRR, Resistance Reserve Ratio; MRR, microvascular resistance reserve.
